# Evidence of transfer of antimicrobial resistance genes from the porcine pathogen *Streptococcus suis* to human clinical isolates of *Streptococcus agalactiae* in a major pig-producing region of Spain

**DOI:** 10.1016/j.onehlt.2026.101396

**Published:** 2026-03-28

**Authors:** Cristina Uruén, María José Lavilla, Virginie Libante, Clara M. Marín, Antonio Rezusta, Sophie Payot, Jesús Arenas

**Affiliations:** a Unit of Microbiology and Immunology, Department of Animal Pathology, Faculty of Veterinary, University of Zaragoza, Spain; b Institute Agrofood of Aragón-IA2, University of Zaragoza-CITA, Zaragoza, Spain; c Department of Microbiology and Parasitology, University Hospital Miguel Servet, Zaragoza, Spain; d IIS Aragón, Zaragoza, Spain; e Université de Lorraine, INRAE, DynAMic, F-54000 Nancy, France; f Department of Animal Science, Centre of Research and Technology of Aragón (CITA), Zaragoza, Spain

**Keywords:** *Streptococcus suis*, Antibiotic resistance, Mobile genetic elements, Inter-species conjugation, Integrative and conjugative elements

## Abstract

The emergence of antimicrobial resistance (AMR) is a global threat, with livestock antibiotic use proposed as a significant contributor. We investigated *Streptococcus suis,* a multidrug-resistant porcine pathogen, as a potential source of AMR gene dissemination to human-associated streptococci in Aragón, Spain. Among 91 porcine isolates recovered across Spain, erythromycin and tetracycline resistance was linked to *erm*(B) and *tet*(O) genes, frequently co-located on Tn*5252*-family integrative and conjugative elements (ICEs) and integrative and mobilizable elements (IMEs). Tn*5252*-family ICEs shared 32–100% of their sequence and carried different AMR gene combinations. Screening of 2388 human clinical streptococcal isolates of different species obtained in Aragón revealed ∼20% erythromycin resistance, particularly *S. pneumoniae* and *S. agalactiae*. Analysis of *erm*(B)-*tet*(O) linkage in a set of erythromycin-resistant isolates and subsequent genome sequencing, revealed twelve isolates carrying Tn5252-family ICEs with both AMR genes. Eight *S. agalactiae* isolates harbored Tn*5252*-family ICEs with >95% nucleotide identity across >91% of their sequence compared with two different ICEs identified in *S. suis* isolates recovered in the same region and period. Conjugation assays confirmed ICE transfer from *S. suis* to *S. agalactiae, S. pneumoniae*, and *S. pyogenes*, while *in vitro* experiments demonstrated that recombination between ICEs promotes diversification of AMR gene cargo within ICEs. Our results identify *S. suis* as a reservoir and generator of ICEs carrying distinct AMR gene combinations that can be horizontally transferred to other human pathogenic streptococci, leading to multidrug resistance in a single step. These findings provide strong evidence supporting interspecies AMR dissemination in regions with intensive pig production and underscore the need for integrated One Health strategies combining veterinary and clinical genomic surveillance, and targeted interventions to limit the spread of mobile genetic elements across species.

## Graphical abstract

**Figure:**
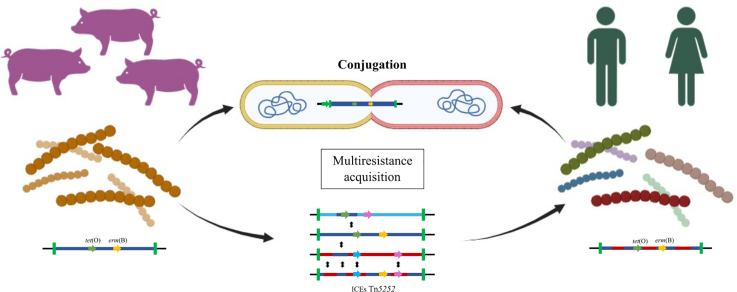

## 1 Introduction

The emergence and spread of antimicrobial resistance (AMR) are major global health challenges. Extensive antibiotic use in animal production may contribute to AMR dissemination by selecting resistant zoonotic bacteria and facilitating gene transfer to human pathogens [1], [2]. Therefore, identifying the bacterial species and mechanisms involved in this transfer is essential for designing effective strategies limiting the spread of AMR from veterinary to human settings.

*Streptococcus suis* is a Gram-positive commensal of porcine upper respiratory tract. Under certain conditions, it can cause sepsis and meningitis [3]. Up to 100% of pigs can harbour *S. suis* on a farm, and over 60–80% of European pig farms are affected [4]. Due to the lack of effective vaccines, antibiotics remain the primary treatment, representing 5–20% of antibiotic use in pig farming [4]. As a commensal, *S. suis* is exposed to antibiotics targeting other pathogens, which has driven high AMR rates globally [5], [6]. *S. suis* can also colonize humans, with carriage rates up to 20% in high-risk populations [7]. Moreover, it is an emerging zoonotic pathogen causing sepsis and meningitis, with recent European incidence estimates ranging from 0.1 to 4.9 cases per 100.000 persons in the at-risk population [8].

*S. suis* harbors diverse mobile genetic elements (MGEs), including prophages, plasmids, transposons, integrative conjugative elements (ICEs), and integrative and mobilizable elements (IMEs), which facilitate the acquisition and dissemination of AMR genes [5], [9]. Comparative analyses have suggested horizontal transfer of AMR genes between *S. suis* and other bacteria [6], [10]. However, most evidence has been derived from *in vitro* experiments and genomic comparisons, leaving uncertainty about whether such gene transfers occur in real-world epidemiological settings. Considering its high prevalence in pigs, the widespread of AMR, and its capacity to colonize humans and other species, this study aimed to investigate the role of *S. suis* in the emergence of AMR in human-pathogenic streptococci and to elucidate underlying mechanisms. We hypothesized that if this process occurs, it is most likely to take place in regions with intensive pig production. Spain is the largest pig producer in Europe, with Aragón as its leading pig-producing region. Therefore, it was chosen as the focus of our study.

## 2 Material and methods

### 2.1 Bacterial and growth conditions

The isolates used and produced in this study are listed in Table S1 and summarized in Text S1. The collection includes 91 *S. suis* clinical isolates previously described obtained from 11 autonomous communities in Spain (2014–2020), representing 10 serotypes and 36 Sequence Types (ST) by multi locus sequence typing (MLST) [11], [12]. The *S. suis* reference strain P1/7 [13] and its fluorescent spectinomycin-resistant mutant derivative P1/7∆g*fp+* [14] were also included. In addition, 2423 clinical isolates from *S. pneumoniae* (*n* = 539), *S. agalactiae* (*n* = 1519), and *S. pyogenes* (*n* = 365) recovered from human patients at Miguel Servet University-Hospital (Zaragoza, Spain) between 2019 and 2021, were included. These strains were identified using MALDI-TOF MS and API Strep (BioMérieux), as part of routine diagnostic procedures. As part of this study, several mutants were generated, and they are described in next sections. Specifically, these included 4 spontaneous rifampicin resistant mutants, Ss_45r, Ss_124r, SagS1R1, SpyS1R1, derived from Ss_45, Ss_124, SagS1R1, SpyS1R1, respectively, a P1/7 transconjugant P1/7ICE115, derived from P1/7, and its derivative mutant P1/7ICE115∆*tetO*, and 6 transformants derived from Ss_45 and Ss_124 (Ss_45r_Tf25, Ss_45r_Tf29, Ss_45r_Tf33, Ss_124r_Tf5, Ss_124r_Tf8, and Ss_124r_Tf10). *S. suis* isolates were grown in Todd-Hewitt Broth (THB, Oxoid) with 15% Agar (THA), while for the remaining streptococcal species, 5% Sheep Blood was added to the medium. All strains were incubated in a candle jar at 37 °C for 24 h. For bacterial liquid cultures, the bacteria were propagated in THB, starting with an Optical Density at 600 nm (OD_600_) of 0.05 as described [11].

### 2.2 Antibiotic resistance determination

Antimicrobial susceptibility to penicillin, clindamycin, erythromycin and tetracycline was evaluated on all clinical strains obtained from human patients and several derivative mutants according to EUCAST recommendations [15], using disk diffusion (Oxoid®) and the microdilution method with the automated Microscan WalkAway (Beckman Coulter) system that enables the determination of the minimal inhibitory concentration.

### 2.3 Whole genome sequencing and bioinformatics

Strains resistant to tetracycline and erythromycin and carrying the genes *tet*(O) and *erm*(B) in *S. suis* and *S. agalactiae* were selected for genome sequencing. For *S. suis*, the size of the PCR amplicon spanning the *tet*(O) and *erm*(B) loci was used as an additional selection criterion, with the aim of capturing a diverse set of MGEs. Together, 11 *S. suis* isolates (Ss_27, Ss_31, Ss_50, Ss_61, Ss_64, Ss_81, Ss_105, Ss_110, Ss_146, Ss_160, and Ss_165) and 11 *S. agalactiae* isolates (Sa_26, Sa_37, Sa_44, Sa_48, Sa_56, Sa_75, Sa_79, Sa_82, Sa_83, Sa_85, and Sa_86) were selected, and their chromosomal DNA was extracted using the Wizard® Genomic DNA Purification Kit (Promega, USA). DNA-Seq library preparation and sequencing were performed at STAB Vida Lda (Caparica, Portugal) using Illumina. Chromosomal DNA from the transformants was extracted with same kit but sequenced at Plasmidsaurus Inc. (Louisville, USA) using nanopore. Raw-read processing and genome assembly were performed as previously described [11], [12]. Additionally, 23 previously published *S. suis* genomes (Ss_02, Ss_08, Ss_20, Ss_45, Ss_46, Ss_52, Ss_53, Ss_69, Ss_70, Ss_72, Ss_84, Ss_92, Ss_93, Ss_100, Ss_106, Ss_109, Ss_115, Ss_121, Ss_124, Ss_134, Ss_156, Ss_166, and Ss_167) were included [11], [12]. To check genome quality dRep bioinformatic tool (3.5.0 version) was used [16], applying the check-M step to evaluate the contamination and completeness of the genomes [17]. The threshold for contamination was 0–5% and for completeness 99–100%. An additional quality criterium proposed by [18] was applied (N50 > 30 kb). STs were determined using MLST 2.0 software [19]. AMR genes were identified with ABRicate using ResFinder and NCBI AMR gene database with a minimum of 80% of DNA identity and DNA coverage. ICEs and IMEs were identified with ICEScreen v1.3.1 [20] using pseudochromosomes as input [9] and were manually delimited in Geneious Prime version 2024.0.7 (Dotmatics). Comparative analyses of MGEs were performed with MAUVE [21]. MGEs were illustrated using EasyFig [22]. Prophages were detected with Phold v0.2.0. Recombination breakpoints were analyzed using RDP4, only recombination events positive to three of the seven detection methods applied (RDP, GENECONV, BootScan, MaxChi, Chimaera, 3Seq, and SiScan) and with a *p*-value <0.05 were considered significant [23].

### 2.4 Genetic constructions and preparation of mutants

Spontaneous rifampicin-resistant mutants, generated in *S. suis* strains Ss_45 and Ss_124, *S. agalactiae* strain SagS1, and *S. pyogenes* strain SpyS1 were performed as described [24] (extended in Text S1**)**. For directed mutagenesis, overlapping PCR was used [25], using the PCR reactions described below. The three amplicons were purified with the FavorPrep™ GEL/PCR Purification Kit (Favorgen, Taiwan) and fused using the In-Fusion® HD Cloning Kit (Takara, Korea). The resulting hybridised fragment was used to transform *S. suis* with the ComS peptide [26], [27]. Transformants were selected on THA plates supplemented with the appropriate antibiotics and incubated at 37 °C under 5% CO_2_ for 24–48 h.

### 2.5 Co-incubation experiments

Mating experiments were performed as reported [28] (detailed in Text S1**)**. The *S. suis* strains Ss_20 and Ss_115 were used as donors; these strains were sensitive to spectinomycin, penicillin, and rifampicin, but resistant to erythromycin and tetracycline. The recipient strains were the spectinomycin-resistant *S. suis* P1/7∆*gfp* + (Saralegui et al., under revision), which is sensitive to erythromycin and tetracycline, the penicillin-resistant *S. pneumoniae* SpS1, and the spontaneous rifampicin-resistant *S. agalactiae* SaS1R1 and *S. pyogenes* SpyS1R2. To assess recombination between ICEs, the resulting transformants were tested by PCR as described below. The conjugation rate was estimated by dividing the number of colony-forming units (CFU) of the transconjugants by the CFU of the donor bacteria at the end of the conjugation experiments. For growth competition assays, the same mating method was used (further described in Text S1**)**. Bacteriocin production was tested as described [28].

### 2.6 PCR amplification

PCR was used for four purposes in this study. First, to detect and *co*-localize the *erm*(B) and *tet*(O) genes using the Supreme NZYLong DNA polymerase kit (Nzytech, Portugal). Second, to discriminate between *Streptococcus* species during mating experiments by targeting species-specific genes, including *gdh* for *S. suis* [29], *mecA* for *S. agalactiae*, *lytA* for *S. pneumoniae* [30], and *spy* for *S. pyogenes* [31]. In *S. suis-S. suis* matings, the spectinomycin-resistance cassette was amplified to identify P1/7Δ*gfp*+ [32]. Third, primers targeting ICE-specific regions (ICE45_rec_D, ICE124_rec_D, and ICE_rec_U) were used to analyse transformants. For the second and third purposes, a standard DNA Taq polymerase kit was used (Biotools, Spain). Finally, PCR was employed to generate directed mutants by replacing *tet*(O) with the *cat gene* using the High-Fidelity Phusion DNA polymerase (Thermo Fisher Scientific, USA). The primers are listed in Table S2. Each PCR reaction contained 0.4–0.5 μM of each primer, 200–500 μM dNTPs, 0.4–1 U of DNA polymerase, and the corresponding buffer. Amplification consisted of initial denaturation at 94 °C for 2–5 min, 35 cycles of 94 °C for 45 s, annealing the primer-specific temperature for 45 s (Table S2), and extension at 68–72 °C for 1 min/kilobase, followed by a final extension at 68–72 °C for 7–30 min. PCR products were separated on 0.7%–1% agarose gels stained with Green®Nucleic Acid Stain (Sigma-Aldrich, Germany), and sequenced at STABVida when required.

## 3 Results

### 3.1 Clinical *S. suis* isolates carry a diversity of MGEs

**Fig. 1:**
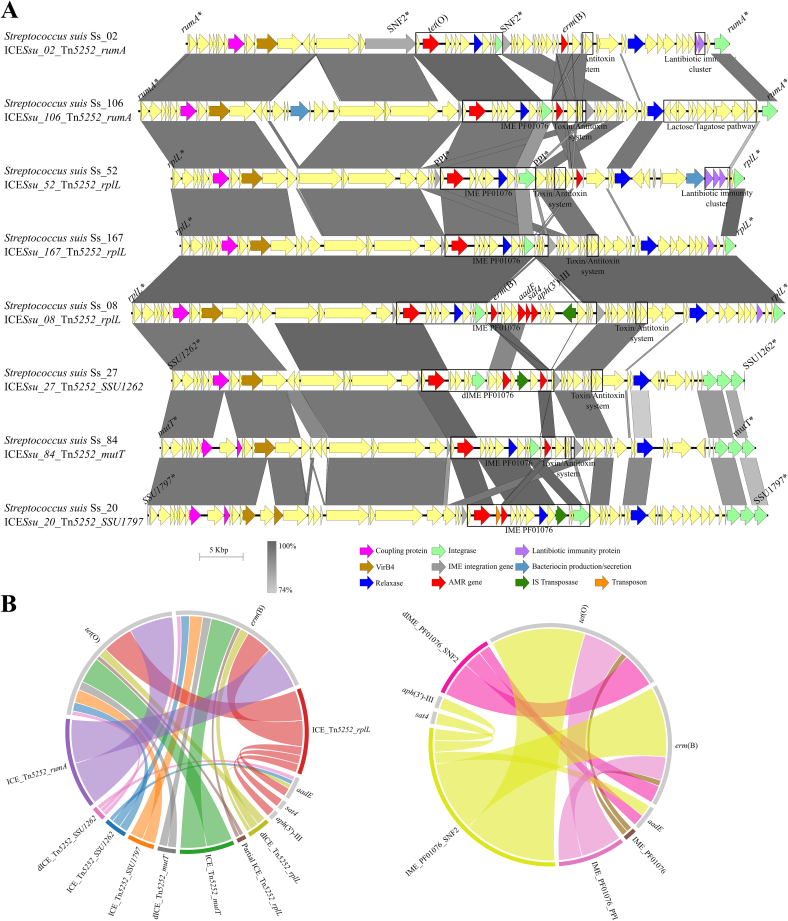
Comparison of the Integrative and Conjugative Elements (ICEs) in *S. suis* isolates belonging to the Tn*5252*-family. (A) Nucleotide comparative in 8 representative ICEs. Arrows represent genes and their orientation within the ICE sequence. Relevant genes involved in the conjugation and integration are shown in different colours, and AMR genes are indicated in red, lantibiotic immunity proteins in violet, bacteriocin production genes in light blue, IS transposases in dark green, and transposons in orange, as declared in the inset. Grey arrows represent the integration genes of Integrative and Mobilizable Elements (IMEs). Similar regions and percentages of identity between ICEs are indicated below in grayscale. Integration sites are specified in the names of the ICEs. (B) AMR genes (grey) carried by the different types of mobile genetic elements (coloured) detected in *S. suis* isolates. ICEs (left panel) and IMEs (right panel) are shown in different graphs. Ribbons are wider when more elements contain the AMR gene. (For interpretation of the references to colour in this figure legend, the reader is referred to the web version of this article.)

Our previous analysis of invasive Spanish swine *S. suis* isolates collected across Spain revealed high resistance rates (>90%) to tetracyclines, macrolides, and lincosamides, which were statistically associated with the *tet*(O) and *erm*(B) genes [12]. Both genes are often co-located on MGEs, mostly ICEs or IMEs, transferable by conjugation [10], [33]. Because our study aimed to demonstrate the transfer of AMR genes to other human-pathogenic species in natural environments, we focused on *tet*(O) and *erm*(B), given their high prevalence and potential mobility. PCR screening of 91 *S. suis* isolates positive for *tet*(O) and *erm*(B) confirmed their co-localization in 79 isolates, with amplicon sizes ranging from 3 kb to 20 kb (Table S3), suggesting that these genes are often carried on the same MGE. Then, genome sequencing of 34 positive isolates from different geographic origins and STs identified a total of 134 MGEs (Table S4 and expanded in Text S1). These included 62 ICEs from four families: Tn*5252* (75.8%), Tn*1549* (14.5%), Tn*GBS2* (8.1%), and ICE*St3* (1.6%), and 87 IMEs from six families: PF01076 (35.6%), PF02486 (32.2%), PF01719 (12.6%), PF01719-PF00910 (8%), PHA00330 (6.9%), and PF13814 (4.6%). AMR genes were detected in 28 ICEs, 5 defective ICEs (dICEs), one partial ICE, 24 IMEs, and 6 defective IMEs (dIMEs) located within ICEs (Table 1). The ICEs/dICEs harbored up to six distinct AMR gene patterns, differing in order, orientation and content (Fig. 1B) while IMEs showed a similar gene composition. All AMR-carrying ICEs belonged to the Tn*5252*-family, and were inserted into *rplL* (29.4%), *rumA* (29.4%), *mutT* (23.5%), ADP ribose pyrophophatase (8.8%) or NTP pyrophosphohydrolase (8.8%) genes (Fig. 1A). AMR-carrying IMEs belonged to the PF01076 family, inserted into SNF2 (22/30) or peptidylprolyl isomerase (PPI) (7/30) genes. Comparative analysis of the Tn*5252*-ICEs harbouring *tet*(O) and/or *erm*(B) revealed 95.4–99.9% nucleotide identity over 32–100% of their sequence length, with genetic distance ranging from 0.33 to 0.99. 22 ICEs showed a genetic distance >0.85, while 12 ICEs were more divergent (<0.85). These findings highlight that *S. suis* harbors diverse MGEs, but those carrying *tet*(O) and *erm*(B) are exclusively Tn*5252*-family ICEs. Although these ICEs share extensive conserved regions also exhibit substantial sequence and gene cargo variability.

### 3.2 Tn5252-family ICEs are present in other streptococci isolated in Spain

Aragón is a leading region in Spanish pig production producing around 40 million pigs/year between 2016 and 2021, while hosting 1.3 million people. Thus, this region is a suitable environment to detect whether AMR gene transfer occurred between *S. suis* and other human pathogens. To investigate this, we analyzed tetracycline and erythromycin resistance in clinical streptococci recovered at the region's major Hospital (Hospital Universitario Miguel Servet, Zaragoza) between 2019 and 2021 (matching the *S. suis* collection period), including 539 *S. pneumoniae,* 1519 *S. agalactiae,* and 365 *S. pyogenes* isolates. *S. pneumoniae* exhibited moderate AMR rates to both antibiotics (15–30%), while *S. agalactiae* revealed higher AMR rates to tetracycline (>30%), and *S. pyogenes* exhibited low AMR rates to all antibiotics tested (Table S5).

**Fig. 2:**
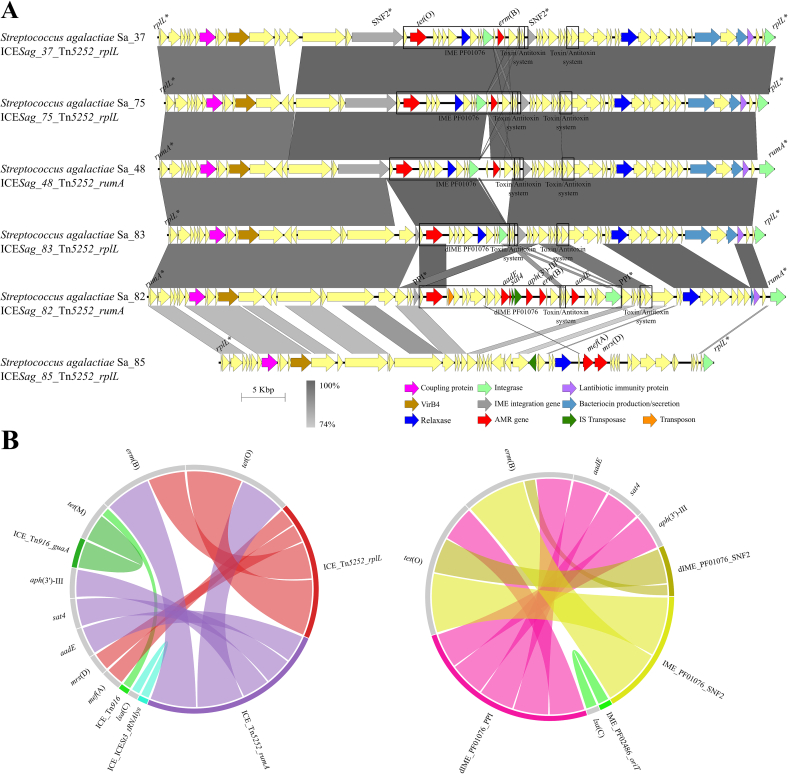
(A) Comparison of the genetic organization of six representative Integrative and Conjugative Elements (ICEs) of the Tn*5252*-family identified in *S. agalactiae* isolates carrying antibiotic resistance (AMR) genes. Colour coding for genes is the same used in Fig. 1. Similar regions and percentages of identity between ICEs are indicated below in grayscale. Integration sites are specified in the names of the ICEs. (B) AMR genes (grey) carried by the different types of mobile genetic elements (coloured) detected in *S. agalactiae* isolates. ICEs (left panel) and IMEs (right panel) are shown in different graphs. Ribbons are wider when more elements contain the AMR gene.

**Fig. 3:**
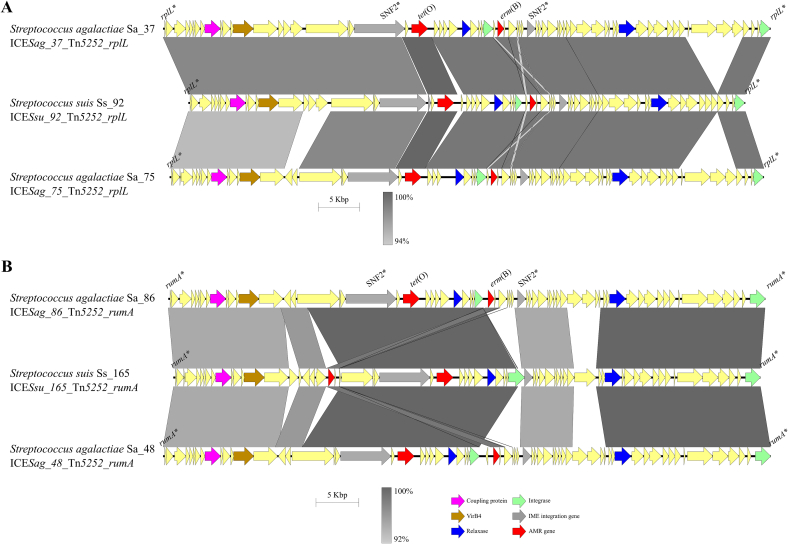
Comparison of most alike Integrative and Conjugative Elements (ICEs) carrying *tet*(O) and *erm*(B) found between *S. suis* and *S. agalactiae* isolates from Aragón. The comparison includes (A) the *S. suis* ICEs with the highest percentage of identity to *S. agalactiae* ICEs, including ICE*Sag_37*_Tn*5252*_*rplL*, ICE*Ssu_92*_Tn*5252*_*rplL*, and ICE*Sag_75*_Tn*5252*_*rplL*, and (B) ICEs with an intermediate percentage of identity, such as ICE*Sag_86*_Tn*5252*_*rumA*, ICE*Ssu_165*_Tn*5252*_*rumA*, and ICE*Sag_48*_Tn*5252*_*rumA*. Colour coding for genes is the same used in Fig. 1. Similar regions and percentages of identity between ICEs are indicated below in grayscale.

PCR screening of representative resistant *S. agalactiae* and *S. pneumoniae* isolates for *tet*(O) and *erm*(B) evidenced 25% of *S. pneumoniae* isolates carried *erm*(B), and 48% and 52% of *S. agalactiae* isolates carried both genes, respectively (Table S1). Eleven *S. agalactiae* carried both genes co-located, presumably within the same MGE (Table S3, Fig. 2A, and extended in Text S1). These isolates belonged to five unrelated STs: ST498 (Sa_26, Sa_37, Sa_44), ST17 (Sa_56, Sa_82, Sa_85), ST28 (Sa_48 and Sa_86), ST529 (Sa_79, and Sa_83), and ST196 (Sa_75). Genome sequencing and MGEs analysis identified 36 ICEs from 6 families: Tn*5252* (36.1%), ICE*St3* (33.3%), Tn*GBS2* (13.9%), Tn*916* (11.1%), Tn*1549* (2.8%), and Tn*GBS1* (2.8%), of which Tn*5252*, Tn*916* and ICE*St3* carried AMR genes. Thirty-nine IMEs from six families were identified at different frequencies: PF01719 (28.9%), PF01076 (26.3%), PF02486 (23.7%), PF01076_PF02486 (7.9%), PHA00330 (7.9%), and PF02407 (5.3%). Twelve IMEs contained AMR genes, corresponding to PF01076 and PF02486 families(Fig. 2B). The *tet*(O) and *erm*(B) genes were identified in 11 Tn*5252-*family ICEs inserted into *rplL* or *rumA* genes. Comparative analysis of these Tn*5252*-ICEs showed a 96.5–99% nucleotide identity over 67–100% of their length (Fig. 2A) and genetic distance ranging from 0.61 to 0.99. Isolates Sa_26, Sa_37 and Sa_44 (ST498) carried identical ICEs suggesting vertical transmission. These findings show that *S. agalactiae* harbors Tn*5252*-family ICEs with *tet*(O)-*erm*(B), as the *S. suis* collection. Comparative analysis of Tn*5252*-family ICEs found in both species revealed different levels of genetic proximity and sequence identify. Interestingly, ICE*Ssu_92*_Tn*5252*_*rplL* of *S. suis* Ss_92 showed 98.6% of sequence identity over 64,984 bp (99.8% of its length) with ICE_Tn*5252*_*rplL* in *S. agalactiae* Sa_26, Sa_37, and Sa_44 (Fig. 3A), and 98% identity over 61,590 bp (94.6% of its length) with ICE_Tn*5252*_*rplL* in Sa_75 (Fig. 3A). Other Tn*5252*-ICEs of *S. suis* isolates such as Ss_31, Ss_165 and Ss_124 showed slightly lower identity (97.5–97.9%) with Tn*5252*-ICEs of *S. agalactiae* isolates Sa_86, Sa_48, and Sa_26 compared with the previous ones (Fig. 3B). Together, at least six of eleven *S. agalactiae* isolates carried Tn*5252*-family ICEs with substantial sequence identity (96.8–98.6%) over >91% of the element compared with ICEs found in *S. suis*. Notably, the most similar ICEs were identified in *S. suis* and *S. agalactiae* isolates recovered in Aragón, suggesting interspecies horizontal transfer of these elements and subsequent diversification.

### 3.3 *S. suis* can transfer AMR genes *via* MGEs to other Streptococcus species *in vitro*

**Fig. 4:**
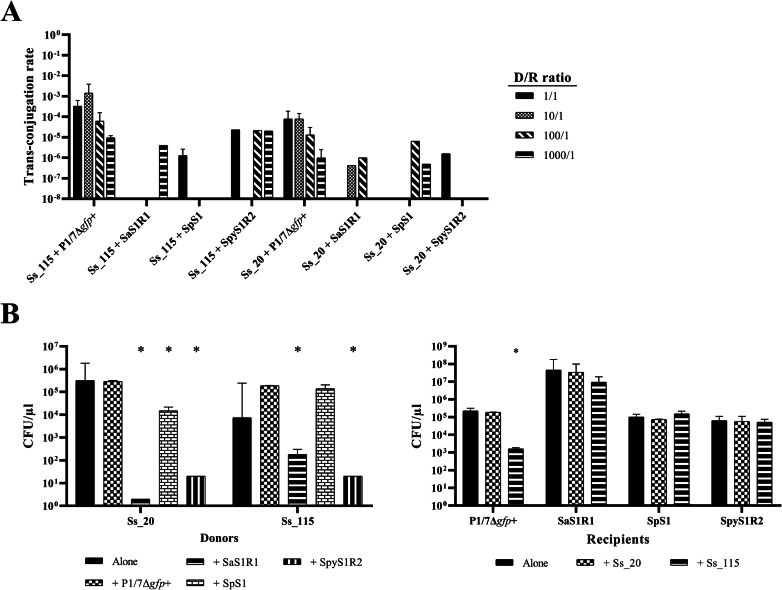
Mating experiments with different *Streptococcus* species. (A) Conjugation rates obtained from mixtures with donor strains Ss_20 and Ss_115 of *S. suis* with recipient strains P1/7Δ*gfp* + of *S. suis*, SaS1R1 of *S. agalactiae*, SpS1 of *S. pneumoniae*, and SpyS1R2 of *S. pyogenes* in 4 different donor/recipient (D/R) ratios. (B) Post-incubation strains quantification. Left panel shows bacterial counts after incubation alone or with donor and recipient strains. Right panel shows the recipient bacterial counts. Data are the median and range of three independent assays. Significant differences (*p* < 0.05, unpaired *t-*test) as compared to the control group (alone) are indicated with one asterisk.

*S. pneumoniae* and *S. pyogenes* lacked the *erm*(B) and *tet*(O) commonly found Tn*5252*-family ICE in *S. suis*, suggesting less frequent AMR gene exchange between these streptococci than with *S. agalactiae*. To investigate the potential of *S. suis* to transfer MGEs, we conducted mating experiments using various donor/recipient ratios. Donor strains were *S. suis* isolates Ss_20 and Ss_115, which harbour ICE_Tn*5252* inserted into the SSU1797 and SSU1262 locus, respectively. Recipient strains included isolates from *S. suis* P1/7Δ*gfp+*, *S. agalactiae* SaS1R1, *S. pneumoniae* SpS1, and *S. pyogenes* SpyS1R2. Conjugation rates varied significantly depending on the recipient species and the donor/recipient ratio (Fig. 4A), obtaining higher conjugation rates when P1/7Δ*gfp* *+* was recipient. To explore whether interspecies-growth competition caused these differences, CFUs of all strains were determined after individual and co-incubated cultures. CFUs of Ss_20 and Ss_115 were significantly (*p* < 0.05) reduced when co-incubated with other streptococcal species, except for Ss_115 with SpS1 (Fig. 4B). Conversely, CFUs of recipients were unaffected, except for P1/7Δ*gfp* *+* co-incubated with Ss_115 (Fig. 4B). No bacteriocin-mediated growth inhibition was observed in any case. The prophage of Ss_20 and three prophages of Ss_115 encode holin products with lytic function. These prophages might be activated during co-incubation, causing donor cell death. Our results demonstrate that *S. suis* can transfer MGEs to other streptococci *in vitro*, although conjugation efficiency is recipient-dependent.

**Table 1:** Characteristics of Mobile Genetic Elements (MGEs) carrying Antimicrobial Resistance (AMR) genes in the genomes of 34 *S. suis* and 11 *S. agalactiae* strains. AMR genes indicated in the [3′-5′] direction is marked with an asterisk, genes that are partial are marked with two asterisks. Integration site of MGEs is indicated at the end of the given name and unidentified integration sites in the genome sequence are denoted with a ^#^ symbol.

Isolate	Name of ICE	VirB4 hit	Integrase(s)	Relaxase family	Size of ICE (bp)	AMR genes in ICE	Name of nested IME	Integrase of IME	Relaxase family of IME	AMR genes in IME
***S. suis***										
**Ss_02**	ICE*Ssu_02*_ Tn*5252*_*rumA*	AER19616	Serine recombinase (AGG64091)	MOBP (PF03432)	62,887	*tet*(O), *erm*(B)	dIME*Ssu_02*_ PF01076_SNF2	Serine recombinase shorter	MOBV (PF01076)	*tet*(O)
**Ss_08**	ICE*Ssu_08*_ Tn*5252*_*rplL*	ADV69676	Tyrosine integrase (ABP92066)	MOBP (PF03432)	75,532	*tet*(O), *erm*(B), *aadE*, sat4, *aph*(3′)-III	IME*Ssu_08*_ PF01076_SNF2	Serine recombinase (ABF36192)	MOBV (PF01076)	*tet*(O), *erm*(B), *aadE*, sat4, *aph*(3′)-III
**Ss_20**	ICE*Ssu_20*_ Tn*5252*_ *SSU1797*	ADV69676	Triplet of serine recombinases (AGZ23088, AGZ23089, AGZ23090)	MOBP (PF03432)	71,711	*tet*(O), *erm*(B)	IME*Ssu_20*_ PF01076_PPI	Serine recombinase (ABF36192)	MOBV (PF01076)	*tet*(O), *erm*(B)
**Ss_27**	ICE*Ssu_27*_ Tn*5252*_ *SSU1262*	ABP89935	Triplet of serine recombinases (AGZ23088, AGZ23089, AGZ23090)	MOBP (PF03432)	66,279	*tet*(O), *aadE*, *erm*(B)	dIME*Ssu_27*_ PF01076_SNF2	–	–	*tet*(O), *aadE*, *erm*(B)
**Ss_31**	ICE*Ssu_31*_ Tn*5252*_*rumA*	AER19616	Serine recombinase (AGG64091)	MOBP (PF03432)	55,690	*tet*(O), *erm*(B)	–	–	–	–
**Ss_45**	ICE*Ssu_45*_ Tn*5252*_*rumA*	ADV69676	Serine recombinase (AGG64091)	MOBP (PF03432)	6881	*erm*(B)*, *tet*(O), *erm*(B)	IME*Ssu_45*_ PF01076_SNF2	Serine recombinase (ABF36192)	MOBV (PF01076)	*tet*(O), *erm*(B)
**Ss_46**	ICE*Ssu_46*_ Tn*5252*_*rumA*	ADV69676	Serine recombinase (AGG64091)	MOBP (PF03432)	11,701	*tet*(O), *erm*(B)	IME*Ssu_46*_ PF01076_PPI	Serine recombinase (ABF36192)	MOBV (PF01076)	*tet*(O), *erm*(B)
**Ss_50**	dICE*Ssu_50*_ Tn*5252*_*mutT*	Partial	Triplet of serine recombinases (AGZ23088, AGZ23089, AGZ23090)	MOBP (PF03432)	64,633	*tet*(O), *erm*(B)	IME*Ssu_50*_ PF01076_SNF2	Serine recombinase (ABF36192)	MOBV (PF01076)	*tet*(O), *erm*(B)
**Ss_52**	ICE*Ssu_52*_ Tn*5252*_*rplL*	ABP89935	Tyrosine integrase (CAZ51585)	MOBP (PF03432)	66,330	*tet*(O), *erm*(B)	IME*Ssu_52*_ PF01076_PPI	Serine recombinase (AER15058)	MOBV (PF01076)	*tet*(O)
**Ss_53**	dICE*Ssu_53*_ Tn*5252*_*rplL*	ABP89935	Tyrosine integrase (ABP92066)	MOBP (PF03432)	65,433	*tet*(O), *erm*(B)	IME*Ssu_53*_ PF01076_PPI	Serine recombinase (AER15058)	MOBV (PF01076)	*tet*(O)
**Ss_61**	ICE*Ssu_61*_ Tn*5252*_*rplL*	ABP89935	Tyrosine integrase (CAZ51585)	MOBP (PF03432)	71,742	*tet*(O), *erm*(B)	IME*Ssu_61*_ PF01076_PPI	Serine recombinase (AER15058)	MOBV (PF01076)	*tet*(O)
**Ss_64**	ICE*Ssu_64*_ Tn*5252*_*rumA*	AER19616	Serine recombinase (AGG64091)	MOBP (PF03432)	62,886	*tet*(O), *erm*(B)	dIME*Ssu_64*_ PF01076_SNF2	Serine recombinase shorter	MOBV (PF01076)	*tet*(O)
**Ss_69**	Partial ICE*Ssu_69*_ Tn*5252*_*rplL*	ABP89935	–	–	48,080	*erm*(B), *tet*(O)	IME*Ssu_69*_ PF01076_SNF2	Serine recombinase (AER15058)	MOBV (PF01076)	*tet*(O)
**Ss_70**	dICE*Ssu_70*_ Tn*5252*_*rplL*	Partial	Tyrosine integrase (AER17235)	MOBP (PF03432)	82,849	*tet*(O), aadE*, *erm*(B)	dIME*Ssu_70*_ PF01076_SNF2	Serine recombinase shorter	MOBV (PF01076)	*tet*(O), aadE*, *erm*(B)
**Ss_72**	ICE*Ssu_72*_ Tn*5252*_*rplL*	ADV69676	Tyrosine integrase (CCW38101)	MOBP (PF03432)	68,265	*tet*(O), *erm*(B)	IME*Ssu_72*_ PF01076_SNF2	Serine recombinase (ABF36192)	MOBV (PF01076)	*tet*(O), *erm*(B)
**Ss_81**	ICE*Ssu_81*_ Tn*5252*_*rumA*	AER19616	Serine recombinase (AGG64091)	MOBP (PF03432)	62,887	*tet*(O), *erm*(B)	dIME*Ssu_81*_ PF01076_SNF2	Serine recombinase shorter	MOBV (PF01076)	*tet*(O)
**Ss_84**	ICE*Ssu_84*_ Tn*5252*_*mutT*	AER17274	Triplet of serine recombinases (AGZ23088, AGZ23089, AGZ23090)	MOBP (PF03432)	68,902	*tet*(O), *erm*(B)	IME*Ssu_84*_ PF01076_SNF2	Serine recombinase (ABF36192)	MOBV (PF01076)	*tet*(O), *erm*(B)
**Ss_92**	ICE*Ssu_92*_ Tn*5252*_*rplL*	ADV69676	Tyrosine integrase (ABP92066)	MOBP (PF03432)	65,088	*tet*(O), *erm*(B)	dIME*Ssu_92*_ PF01076_SNF2	Serine recombinase shorter	MOBV (PF01076)	*tet*(O), *erm*(B)
**Ss_93**	ICE*Ssu_93*_ Tn*5252*_ *SSU1797*	ADV69676	Triplet of serine recombinases (AGZ23088, AGZ23089, AGZ23090)	MOBP (PF03432)	66,630	*tet*(O), *erm*(B)	IME*Ssu_93*_ PF01076^#^	Serine recombinase (ABF36192)	MOBV (PF01076)	*tet*(O), *erm*(B)
**Ss_100**	ICE*Ssu_100*_ Tn*5252*_*mutT*	AER17274	Triplet of serine recombinases (AGZ23088, AGZ23089, AGZ23090)	MOBP (PF03432)	66,159	*tet*(O), *erm*(B)	IME*Ssu_100*_ PF01076_SNF2	Serine recombinase (ABF36192)	MOBV (PF01076)	*tet*(O), *erm*(B)
**Ss_105**	ICE*Ssu_105*_ Tn*5252*_*rumA*	ADV69676	Serine recombinase (AGG64091)	MOBP (PF03432)	67,845	*tet*(O), *erm*(B)*	IME*Ssu_105*_ PF01076_PPI	Serine recombinase (AER17248)	MOBV (PF01076)	*tet*(O), *erm*(B)*
**Ss_106**	ICE*Ssu_106*_ Tn*5252*_*rumA*	ADV69676	Serine recombinase (AGG64091)	MOBP (PF03432)	73,848	*tet*(O), *erm*(B)	IME*Ssu_106*_ PF01076_SNF2	Serine recombinase (ABF36192)	MOBV (PF01076)	*tet*(O), *erm*(B)
**Ss_109**	ICE*Ssu_109*_ Tn*5252*_ *SSU1797*	AER15081	Triplet of serine recombinases (AGZ23088, AGZ23089, AGZ23090)	MOBP (PF03432)	70,309	*tet*(O), *erm*(B)	IME*Ssu_109*_ PF01076_PPI	Serine recombinase (ABF36192)	MOBV (PF01076)	*tet*(O), *erm*(B)
**Ss_110**	ICE*Ssu_110*_ Tn*5252*_*mutT*	ADV69676	Triplet of serine recombinases (AGZ23088, AGZ23089, AGZ23090)	MOBP (PF03432)	66,112	*tet*(O), *erm*(B)	IME*Ssu_110*_ PF01076_SNF2	Serine recombinase (ABF36192)	MOBV (PF01076)	*tet*(O), *erm*(B)
**Ss_115**	ICE*Ssu_115*_ Tn*5252*_ *SSU1262*	ADV69676	Triplet of serine recombinases (AGZ23088, AGZ23089, AGZ23090)	MOBP (PF03432)	65,505	*tet*(O), *erm*(B)	–	–	–	–
**Ss_121**	ICE*Ssu_121*_ Tn*5252*_*mutT*	ADV69676	Triplet of serine recombinases (AGZ23088, AGZ23089, AGZ23090)	MOBP (PF03432)	70,401	*erm*(B), *tet*(O)	IME*Ssu_121*_ PF01076_SNF2	Serine recombinase (ABF36192)	MOBV (PF01076)	*tet*(O)
**Ss_124**	ICE*Ssu_124*_ Tn*5252*_*rplL*	AER15081	Tyrosine integrase (ABP92066)	MOBP (PF03432)	75,451	*tet*(O), *erm*(B), *aadE*, *sat4*, *aph*(3′)-III	IME*Ssu_124*_ PF01076_SNF2	Serine recombinase (ABF36192)	MOBV (PF01076)	*tet*(O), *erm*(B), *aadE*, *sat4*, *aph*(3′)-III
**Ss_134**	dICE*Ssu_134*_ Tn*5252*_ *SSU1262*	ABP89935	Triplet of serine recombinases (AGZ23088, AGZ23089, AGZ23090)	MOBP (PF03432)	66,143	*tet*(O), *aadE*, *erm*(B)	–	–	–	–
**Ss_146**	ICE*Ssu_146*_ Tn*5252*_*mutT*	AER17274	Triplet of serine recombinases (AGZ23088, AGZ23089, AGZ23090)	MOBP (PF03432)	66,159	*tet*(O), *erm*(B)	IME*Ssu_146*_ PF01076_SNF2	Serine recombinase (ABF36192)	MOBV (PF01076)	*tet*(O), *erm*(B)
**Ss_156**	ICE*Ssu_156*_ Tn*5252*_*mutT*	AER17274	Triplet of serine recombinases (AGZ23088, AGZ23089, AGZ23090)	MOBP (PF03432)	66,163	*tet*(O), *erm*(B)	IME*Ssu_156*_ PF01076_SNF2	Serine recombinase (ABF36192)	MOBV (PF01076)	*tet*(O), *erm*(B)
**Ss_160**	ICE*Ssu_160*_ Tn*5252*_*rumA*	ADV69676	Serine recombinase (AGG64091)	MOBP (PF03432)	72,867	*tet*(O), *erm*(B)	IME*Ssu_160*_ PF01076_SNF2	Serine recombinase (ABF36192)	MOBV (PF01076)	*tet*(O), *erm*(B)
**Ss_165**	ICE*Ssu_165*_ Tn*5252*_*rumA*	ADV69676	Serine recombinase (AGG64091)	MOBP (PF03432)	68,486	*erm*(B), *tet*(O)	IME*Ssu_165*_ PF01076_SNF2	Serine recombinase (ABF36192)	MOBV (PF01076)	*tet*(O)
**Ss_166**	dICE*Ssu_166*_ Tn*5252*_*mutT*	Partial	Triplet of serine recombinases (AGZ23088, AGZ23089, AGZ23090)	MOBP (PF03432)	92,082	*tet*(O), *erm*(B)	–		–	–
**Ss_167**	ICE*Ssu_167*_ Tn*5252*_*rplL*	ADV69676	Tyrosine integrase (ABP92066)	MOBP (PF03432)	64,316	*tet*(O)	IME*Ssu_167*_ PF01076_SNF2	Serine recombinase (ABF36192)	MOBV (PF01076)	*tet*(O)
***S. agalactiae***										
**Sa_26**	ICE*Sag_26*_ Tn*916*_*guaA*	CBJ22573	Tyrosine integrase (CBJ22584)	MOBT (PF02486)	21,853	*tet*(M)	–	–	–	–
	ICE*Sag_26*_ Tn*5252*_*rplL*	ABP89935	Tyrosine integrase (ADX24462)	MOBP (PF03432)	71,022	*tet*(O), *erm*(B)	IME*Sag_26*_ PF01076_SNF2	Serine recombinase (ABF36192)	MOBV (PF01076)	*tet*(O), *erm*(B)
**Sa_37**	ICE*Sag_37*_ Tn*916*_*guaA*	CBJ22573	Tyrosine integrase (CBJ22584)	MOBT (PF02486)	21,864	*tet*(M)	–	–	–	–
	ICE*Sag_37*_ Tn*5252*_*rplL*	ABP89935	Tyrosine integrase (ADX24462)	MOBP (PF03432)	71,022	*tet*(O), *erm*(B)	IME*Sag_37*_ PF01076_SNF2	Serine recombinase (ABF36192)	MOBV (PF01076)	*tet*(O), *erm*(B)
**Sa_44**	ICE*Sag_44*_ Tn*916*_*guaA*	CBJ22573	Tyrosine integrase (CBJ22584)	MOBT (PF02486)	21,853	*tet*(M)	–	–	–	–
	ICE*Sag_44*_ Tn*5252*_*rplL*	ABP89935	Tyrosine integrase (ADX24462)	MOBP (PF03432)	71,022	*tet*(O), *erm*(B)	IME*Sag_44*_ PF01076_SNF2	Serine recombinase (ABF36192)	MOBV (PF01076)	*tet*(O), *erm*(B)
**Sa_48**	ICE*Sag_48*_ Tn*5252*_*rumA*	ADV69676	Serine recombinase (AGG64091)	MOBP (PF03432)	70,880	*tet*(O), *erm*(B)	IME*Sag_48*_ PF01076_SNF2	Serine recombinase (ABF36192)	MOBV (PF01076)	*tet*(O), *erm*(B)
**Sa_56**	ICE*Sag_56*_ Tn*5252*_*rplL*	CCF02711	Tyrosine integrase (CCW38101)	MOBP (PF03432)	56,990	*mef*(A), *mrs*(D)	–	–	–	–
	ICE*Sag_56*_ Tn*5252*_*rumA*	ADV69676	Serine recombinase (AGG64091)	MOBP (PF03432)	71,171	*tet*(O), *aadE*, sat4**, *aph*(3′)-III, *erm*(B), *aadE*	dIME*Sag_56*_ PF01076_PPI	Serine recombinase (AER15058)	–	*tet*(O), *aadE*, sat4**, *aph*(3′)-III, *erm*(B), *aadE*
**Sa_75**	ICE*Sag_75*_ Tn*5252*_*rplL*	ADV69676	Tyrosine integrase (ADX24462)	MOBP (PF03432)	69,483	*tet*(O), *erm*(B)	IME*Sag_75*_ PF01076_SNF2	Serine recombinase (ABF36192)	MOBV (PF01076)	*tet*(O), *erm*(B)
**Sa_79**	ICE*Sag_79*_ Tn*5252*_*rplL*	ADV69676	Tyrosine integrase (ADX24462)	MOBP (PF03432)	68,772	*tet*(O)	dIME*Sag_79*_ PF01076_SNF2	Serine recombinase shorter	MOBV (PF01076)	*tet*(O)
	ICE*Sag_79*_ ICE*St3*_*tRNAlys*	EAO72173	Tyrosine integrase xerC	MOBT (PF02486)	38,752	*lsa*(C)	IME*Sag_79*_ PF02486_*oriT*	Tyrosine integrase	MOBT (PF02486)	*lsa*(C)
**Sa_82**	ICE*Sag_82*_ Tn*5252*_*rumA*	ADV69676	Serine recombinase (AGG64091)	MOBP (PF03432)	73,408	*tet*(O), *aadE*, sat4**, *aph*(3′)-III, *erm*(B), *aadE*	dIME*Sag_82*_ PF01076_PPI	Serine recombinase (AER15058)	–	*tet*(O), *aadE*, sat4**, *aph*(3′)-III, *erm*(B), *aadE*
**Sa_83**	ICE*Sag_83*_ Tn*5252*_*rplL*	ADV69676	Tyrosine integrase (ADX24462)	MOBP (PF03432)	68,772	*tet*(O)	dIME*Sag_83*_ PF01076_SNF2	Serine recombinase shorter	MOBV (PF01076)	*tet*(O)
**Sa_85**	ICE*Sag_85*_ Tn*5252*_*rplL*	CCF02711	Tyrosine integrase (CCW38101)	MOBP (PF03432)	56,909	*mef*(A), *mrs*(D)	–		–	–
	ICE*Sag_85*_ Tn*5252*_*rumA*	ADV69676	Serine recombinase (AGG64091)	MOBP (PF03432)	73,669	*tet*(O), *aadE*, sat4**, *aph*(3′)-III, *erm*(B), *aadE*	dIME*Sag_85*_ PF01076_PPI	Serine recombinase (AER15058)	–	*tet*(O), *aadE*, sat4**, *aph*(3′)-III, *erm*(B), *aadE*
**Sa_86**	ICE*Sag_86*_ Tn*916*	BAK30694	Tyrosine integrase (EIK41785)	MOBT (PF02486)	18,038	*tet*(M)	–		–	–
	ICE*Sag_86*_ Tn*5252*_*rumA*	ADV69676	Serine recombinase (AGG64091)	MOBP (PF03432)	69,637	*tet*(O), *erm*(B)	dIME*Sag_86*_ PF01076_SNF2	Serine recombinase shorter	MOBV (PF01076)	*tet*(O), *erm*(B)

Abbreviations: SNF2; encoding a putative helicase protein, PPI: peptidylprolyl isomerase, “-“: no IME inserted in ICE.

**Fig. 5:**
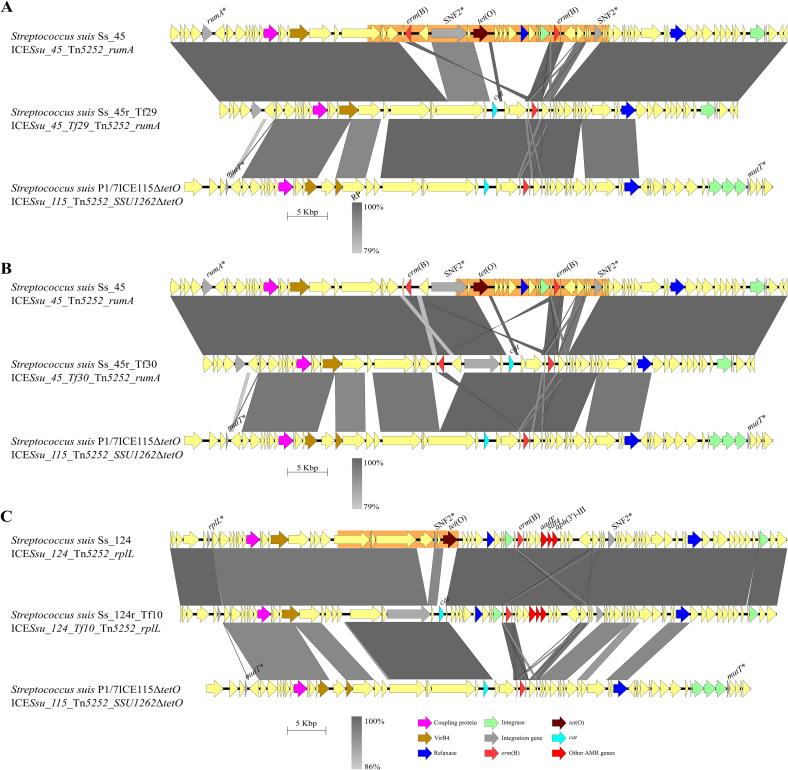
Mosaic organization of Integrative and Conjugative elements (ICEs) generated by recombination within ICEs. Genetic rearrangements (A, B) in the ICE of the transformants Ss_45r (C) and Ss_124r after incubation with P1/7ICE115Δ*tetO*. For all panels, the recipient and donor strain are located above and below the recombinant strain, respectively. Gene colour coding follows the scheme used in Fig. 1, with additional arrows in pink indicating the *erm*(B) gene, maroon arrows indicating *tet*(O), and green arrows indicating the *cat* gene. The most probable recombination area is highlighted in orange. Similar regions and percentages of identity between ICEs are indicated below in grayscale. (For interpretation of the references to colour in this figure legend, the reader is referred to the web version of this article.)

### 3.4 ICE diversity can be generated by recombination events

**Supplementary Fig. S1:**
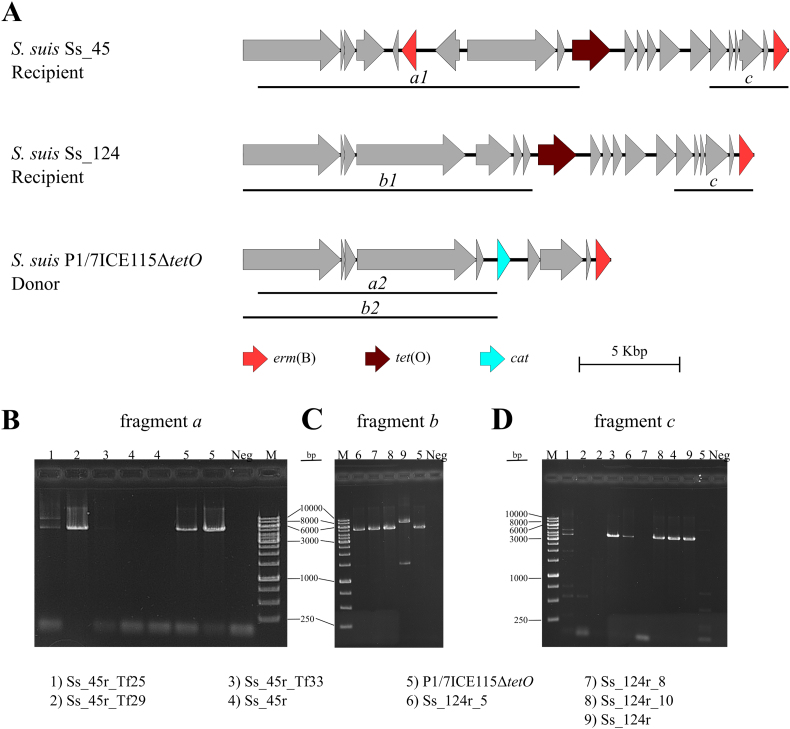
(**A**) Schematic representation of DNA fragments *a*, *b*, and *c* for screening recombinant clones. Portions of the Integrative and Conjugative Element (ICE) from the recipient strains Ss_45r and Ss_124r, and the donor strain P1/7ICE115Δ*tetO,* are shown*.* The expected length of the fragment *a1* is 9338 base pairs (bp) and of the fragment *a2* is 5209 bp. The expected length of the fragment *b1* is 7654 bp and of the fragment *b2* is 5259 bp. The expected length of the fragment *c* in Ss_45r and Ss_124r is of 3707 bp and 3654 bp, respectively. No amplification is expected for this fragment in P1/7ICE115Δ*tetO*. Genes are shown as grey arrows, antimicrobial resistance genes (AMR) are indicated with different colours (see legend). (**B—C**) Agarose gels showing PCR products obtained in different strains using primers for (**B**) fragment *a*, (**C**) fragment *b*, and (**D**) fragment *c*. Templates are labelled as follows: line 1) Ss_45r_Tf25, line 2) Ss_45r_Tf29, line 3) Ss_45r_Tf33, line 4) Ss_45r, line 5) P1/7ICE115Δ*tetO*, line 6) Ss_124r_5, line 7) Ss_124r_8, line 8) Ss_124r_10, and line 9) Ss_124r. Abbreviations: ICE, Integrative and Conjugative Elements. Neg, ultra-pure water. Interpretation: To analyse the downstream-*tet*(O) region, different primers sets were designed for the transformants of Ss_45r and Ss_124r, yielding fragments *a* and *b*. If the fragment length matched that of the recipient strain, the fragments were denominated as *a1* and *b1.* Conversely, if the fragment length matched that of the donor, they were referred as a2 and b2 (Fig. S1A). All Ss_45r transformants yielded fragment *a2* (Fig. S1B). Similarly, all Ss124r transformants yielded fragment *b2* (Fig. S1C). These results suggest that the downstream region in all transformants shared the same organization as the donor strain. In contrast, analysis of the upstream *tet*(O) region (fragment *c*) revealed PCR amplification only in recipient-like sequences (Fig. S1A). All 6 transformants were tested for the presence of fragment *c* (Fig. S1D). Clones Ss_45r_Tf25, Ss_45r_Tf33, Ss_124r_Tf5, and Ss_124r_Tf10 yielded fragment *c,* consistent with the recipient strains, but clones Ss_45r_Tf29 and Ss_124r_Tf8 were negative (Fig. S1D).

Tn*5252*-family ICEs from *S. suis* contain shared regions but display high variability in gene cargo content. We hypothesized this results from recombination events between ICEs. To investigate this, we engineered a mutant in strain P1/7Δ*gfp*+, which harbors ICE*Ssu_115*_Tn*5252*_*SSU1262* (P1/7ICE115), by substituting *tet*(O) with a chloramphenicol-resistance cassette (*cat*) (P1/7ICE115Δ*tetO*). Genomic DNA of this mutant was used to transform rifampicin-resistant isolates Ss_45r and Ss_124r, which harbour ICE*Ssu_45*_Tn*5252*_*rumA* and ICE*Ssu_124*_Tn*5252*_*rplL*, respectively. Three transformants of Ss_45r (Ss_45r_Tf25, Ss_45r_Tf29, and Ss_45r_Tf33) and three from Ss_124r (Ss_124r_Tf5, Ss_124r_Tf8, and Ss_124r_Tf10) were tested by PCR and detailed results are provided in Figs. S1B-C and Text S1. Briefly, PCR screening suggested that the ICE of Ss_45r_Tf25, Ss_45r_Tf33, Ss_124r_Tf5, and Ss_124r_Tf10 had ICEs with upstream- and downstream-*tet*(O) regions identical to the recipient ICE, whereas ICEs of clones Ss_45r_Tf29 and Ss_124r_Tf8 had downstream-*tet*(O) region matching the recipient, but the upstream-*tet*(O) region of donor ICE. Whole-genome sequencing of Ss_45r_Tf29, Ss_45r_Tf33, and Ss_124r_Tf10 confirmed structural differences in the ICEs of the three transformants (Fig. 5A-C detailed in Text S1). Analysis with RDP4 detected significant recombination points (*p*-value of 1 × 10^−30^) in the three ICEs (orange coloured in Fig. 5A-C). Notably, each transformant carried a uniquely composed Tn*5252*-family ICE, demonstrating that distinct recombination events can generate ICE diversity.

## 4 Discussion

While plasmid-mediated spread is a well-established mechanism for AMR dissemination from farms to humans, the contribution of conjugative chromosomal elements is less well understood. *S. suis*, a porcine pathogen highly prevalent and a multidrug-resistant bacterium, is considered a reservoir of AMR genes. This study provides, for the first time, substantial evidence that combines comparative genomics of clinical strains from the same geographic region with *in vitro* conjugation assays, supporting the conclusion that this bacterium transfers AMR genes directly to human-pathogenic streptococci.

Previous studies have proved *in vitro* that *S. suis* can transfer AMR genes to other species, including *S. pyogenes* [34], [35], [36], [37], *S. agalactiae* [37], [38], *S. pneumoniae* [35], [36], *S. oralis* [36], and *S. thermophilus* [39]. These results were confirmed in our work using clinical isolates. We observed higher transfer rates between *S. suis* strains than with other streptococci, consistent with a prior report [35]. However, the efficiency of the transfer was diverse depending on the recipient species and occurred under specific D/R ratios. This indicates that horizontal gene transfer is highly dependent of the context, including the bacterial density and interspecies competition. Furthermore, our results showed reduced viability of donor strains during co-incubation with the recipients, this phenomenon could be caused by inter-strain growth inhibition systems, including bacteriocin production, previously reported in several streptococci species [40], [41], [42], or by the activation of lytic prophages [43]. Additionally, the activity of restriction-modification systems and clustered regularly interspaced short palindromic repeats defences may prevent ICE insertion and reduce transfer efficiency as demonstrated in *S. agalactiae* [44]. Thus, AMR transfer from *S. suis* to other streptococci *via* conjugation may be limited by different factors. This can explain the fact that most previous evidence of interspecies AMR gene transfer involving *S. suis* comes from comparative genomic analyses of clinical isolates rather than direct experimental demonstration in natural environments. As conjugation *in vitro* is hard to reproduce, its frequency *in vivo* it is assumed to be low. For instance, Martel, et al. (2005) [36] detected *erm*(B) and *tet*(O) in *S. suis* isolates and other streptococcal species of both human and animal origin in Belgium, while the *tet*(O/W/32/O) gene was identified in pig isolates of *S. suis* in Italy [45] and in a *S. gallolyticus* isolate from a human patient in Germany [46]. Also, Huang et al. (2016) [10] identified ICEs of the family Tn*5252* carrying *tet* and *erm* gene variants in seven Chinese *S. suis* isolates that were present in streptococcal species deposited in public databases [10]. However, reconciling these comparative analyses with interbacterial interaction required for conjugation is challenging, as these elements show limited sequence homology and ICEs of the same family and AMR genes are widely distributed across streptococci species. Furthermore, clinical isolates and public genomes sharing these elements are geographically very distant. Here, we detected a large diversity of conjugative elements, including Tn*5252*-family ICEs, in our *S. suis* collection. These elements carry *tet*(O) and *erm*(B) but show highly variable lengths and a mosaic genetic pattern. Remarkably, we detected *S. suis* isolates harbouring Tn*5252*-family ICEs almost identical to those found in *S. agalactiae* isolates, and both species were recovered from the same region and time period. To the best of our knowledge, this study shows, for the first time, substantial evidence of transfer between *S. suis* and other species in a within a shared epidemiological context involving both animal and human populations.

*S. suis* can colonize the mucosa of several animal species and behave as a commensal. Previous reports have detected this bacterium in high-risk populations, including pig workers, meat inspectors, veterinarians, slaughterhouse workers, or butchers [47], [48], [49], [50]. While the duration of colonization is unknown, it can persist for at least three weeks [51]. In contrast, *S. agalactiae* is a natural component of the human microbiota. We hypothesize that in Aragón, *S. suis* colonized humans and, by sharing an ecological niche with *S. agalactiae,* transferred AMR genes *via* Tn*5252*-family ICEs. However, we cannot determine whether the exchange occurred directly on these strains or through an intermediate host. A interestingly observation is that three out of the six *S. agalactiae* isolates sharing an ICE with *S. suis* isolates belonged to the same ST but were from different patients. This suggests that a common ancestor acquired the ICE from *S. suis*, with subsequent vertical dissemination. Slight sequence differences indicate that these ICEs evolved independently after transfer. The remaining isolates belonged to different STs and carry ICEs with slightly lower identity, suggesting independent transfer events either from *S. suis* or *S. agalactiae* strains. Additionally, some isolates show a lower identity with ICEs of our *S. suis* collection, probably reflecting older transfer events followed by diversification.

Tn*5252*-family ICEs in *S. suis* revealed a mosaic gene organization with variable AMR genes composition. The origin of the variability in the adaptive module of ICEs was attributed to different mechanisms [52], including recombination, transposon activity, or acquisition of new ICEs. Marini et al. (2015) reported a hybrid ICE generated by recombination between *S. suis* ICE*Ssu32457* and *S. agalactiae* ICE*Sa2603* probably transferred by conjugation [38]. However, conjugation leads, generally, to the acquisition of a complete ICE or duplication. Despite sequence similarity with ICEs present in the recipient strain, site specific recombination is more efficient than homologous recombination, and intracellular intermediates (circled double stranded DNA) are not substrates for RecA. Thus, the acquisition of the ICE rather than recombination will be favored during conjugation. This does not exclude that ICEs can be recognized by self-protecting mechanisms, and the subsequent products can then be recognized by the homologous recombination system. We proposed that transformation rather than conjugation can be a key driver of ICE diversification, likely promoting the diversity found in Tn*5252*-family ICEs of *S. suis* (Fig. 1A). This was proved here by experiments using DNA from strains carrying reporter genes within Tn*5252*-family ICEs and further genome sequencing of recombinants. This phenomenon was also reported in other streptococcal species, including in Tn*916*-related ICEs of *S. pneumoniae* [53], ICE_Tn*5252* of *S. agalactiae* [38], as well as within ICEs of *Enterococcus faecalis* [54]. The resulting hybrids with new gene content were transferred by conjugation to other streptococci, demonstrating that gene exchange did not affect the transfer mechanism, generating thus a new transferable AMR gene combination. Comparative analysis of the frequency of these mechanisms, which was not addressed in this study, would help clarify their relative contribution to AMR diversification.

## 5 Conclusions

*S. suis* harbors a large repertoire of conjugative MGEs, which can carry multiple AMR genes and disseminate multi-drug resistance. The AMR genes patterns within a MGEs is variable and it can be generated through recombination events. These new combinations can eventually be transferred to other streptococcal species, generating multi-resistant strains in a single step. Our findings emphasize the need for integrated One Health approaches to reduce the spread of AMR.

Routine genomic monitoring of mobile genetic elements in both animal and human isolates could enable early detection of cross-species transmission events. In addition, targeted interventions such as antimicrobial stewardship programs in pig production, routine genomic surveillance of mobile genetic elements across veterinary and clinical sectors, or strict biosecurity measures to reduce animal to human transmission should be prioritized.

The following is the supplementary data related to this article.

Supplementary Text S1 Expanded description of material and methods, and results.

Supplementary Table S1 Strains and clinical isolates used in this study.
Abbreviations: ST: Sequence type, ERY: Erythromycin, CLIN: Clindamycin, TET: Tetracycline, NT: Non-typable, AMP: Ampicillin

Supplementary Table S2 Primers used in the study. The size of the expected PCR products and annealing temperature (T_A_) for each PCR reaction is also indicated. Abbreviations: bp: base pairs, T_A_: annealing temperature, AMR: Antimicrobial Resistance.

Supplementary Table S3 Molecular sizes (kb) obtained by PCR for the co-localization of *tet*(O) and *erm*(B) genes in 91 *S. suis* isolates and 11 *S. agalactiae isolates*.

Supplementary Table S4 Mobile Genetic Elements identified in the 34 genomes of *S. suis* and 11 genomes of *S. agalactiae*. The family of the Integrative and Conjugative Elements (ICEs) or Integrative and Mobilizable Elements (IMEs) is indicated for each MGE found in every isolate.

Supplementary Table S5 Prevalence of antimicrobial resistance to different antibiotics in isolates of *S. pneumoniae, S. agalactiae,* and *S. pyogenes* from human patients at Miguel Servet University (Zaragoza, Spain) between 2019 and 2021.

## CRediT authorship contribution statement

**Cristina Uruén:** Writing – review & editing, Writing – original draft, Visualization, Software, Methodology, Investigation, Formal analysis, Data curation. **María José Lavilla:** Writing – review & editing, Methodology, Investigation. **Virginie Libante:** Writing – review & editing, Validation, Supervision, Methodology, Investigation. **Clara M. Marín:** Writing – review & editing, Methodology, Investigation. **Antonio Rezusta:** Writing – review & editing, Methodology, Investigation. **Sophie Payot:** Writing – review & editing, Supervision, Software, Resources, Funding acquisition, Conceptualization. **Jesús Arenas:** Writing – review & editing, Writing – original draft, Supervision, Resources, Project administration, Funding acquisition, Formal analysis, Data curation.

## Ethical approval

No ethical approval is required.

## Funding

This work received funding from Gobierno de Aragón (Department of I + D + I project in priority lines, Grant agreement LMP58_21), and Ministerio de Ciencia e Innovación/Agencia Española de Investigación MCIN/AEI/10.13039/501100011033, as appropriate, by ERDF A way of making Europe by the European Union or by the European Union NextGeneration EU/PRTR (Grant agreements PID2020-114617RB-100 and PID2023-146823OB-I00). The funders had no role in study design, data collection and analysis, decision to publish, or preparation of the manuscript.

## Declaration of competing interest

The authors declare that they have no conflict of interest.

## Data Availability

The datasets used in this study are available online in the database of NBCI under the bioprojects PRJNA1037519 and PRJNA1037513. Sequence of ICEs mutated during this study can be found below: ICE*Ssu_115*_Tn*5252*_*SSU1262*Δ*tetO* (PX363404), ICE*Ssu_45_Tf29*_Tn*5252*_*rumA* (PX363405), ICE*Ssu_45_Tf33*_Tn*5252*_*rumA* (PX363406), and ICE*Ssu_124_Tf10*_Tn*5252_rplL* (PX363407).

## Glossary

AMR: Antimicrobial Resistance
MGE: Mobile Genetic Element
ICEs: Integrative and Conjugative Elements
IMEs: Integrative and Mobilizable Elements
ST: Sequence Type
MLST: Multi-Locus Sequence Type
THB: Todd-Hewitt Broth
THA: THB with 15% of Agar
OD_600_: Optical Density at 600 nm
CFUs: Colony Forming Units
dICE: defective ICE
dIME: defective IME
PPI: Peptidylprolyl isomerase
