# Supplementary material for: Evidence of transfer of antimicrobial resistance genes from the porcine pathogen Streptococcus suis to human clinical isolates of Streptococcus agalactiae in a major pig-producing region of Spain
Source: One Health. 2026 Mar 28;22:101396. doi: 10.1016/j.onehlt.2026.101396 (PMC13089153; doi:10.1016/j.onehlt.2026.101396)
Supplement: Supplementary Text S1 — Expanded description of material and methods, and results. [file mmc2.docx]

**Text S1 Expanded sections**

**Material and Methods**

## ***Bacterial isolates and growth conditions***

All isolates used in this study are listed in **Table S1**. This includes a panel of 91 *S. suis* isolates obtained from sick pigs in Spain during the 2015-2020 period [1, 2]**.** They were obtained from 11 Spanish regions, including Aragón (n= 36), Catalonia (n= 22), Castilla y Leon (n= 12), Castilla La-Mancha (n= 6), Murcia (n= 6), Andalucia (n= 3), Galicia (n= 2), Extremadura (n= 1), Valencia (n= 1), Cantabria (n= 1), and Madrid (n= 1). They belong to 10 serotypes, including serotype 9 (n= 29), serotype 1 (n= 16), serotype 7 (n= 13), serotype 2 (n=9), and 10 were non-typable. They were distributed into 36 STs, comprising ST123 (n= 22), ST1 (n= 17), and ST29 (n= 8). In addition, the *S. suis* reference strain P1/7 [3] of serotype 2 and its fluorescent spectinomycin-resistant mutant P1/7∆g*fp+* were also included. Furthermore, a panel of 2,388 clinical isolates from *S. pneumoniae* (n=529), *S. agalactiae* (n=1,497), and *S. pyogenes* (n=362) recovered from sick human patients at Miguel Servet University Hospital (Zaragoza, Spain) between the 2019-2021 period. Human clinical isolates were identified by MALDITOFF.

## ***Genetic constructions and preparation of mutants***

For spontaneous rifampicin-resistant mutants, bacterial strains were grown overnight in 3 ml of THB for *S. agalactiae,* or in THB supplemented with 5% defibrinated sheep blood for *S. pyogenes* and *S. pneumoniae* at an initial OD_600_ of 0.05. The bacteria were then harvested by centrifugation (4000 *g* during 10 min), resuspended in THB, and plated onto THA supplemented with different rifampicin concentrations (25, 50, and 75 mg/L). Cultures were incubated at 37°C under 5% CO_2_ for 24-48 h. Spontaneous rifampicin-resistant colonies were sub-cultured onto a fresh THA plate containing 100 mg/L of rifampicin to confirm resistance.

For directed mutagenesis, overlapping PCR was used following a previously described strategy [4]. Briefly, three DNA fragments consisting of an upstream and downstream flanking sequence of the gene of interest and an AMR-cassette were obtained through separate PCR reactions. The bands obtained that corresponded to the target fragments were excised from 0.7% agarose gel and purified using the FavorPrep^TM^ GEL/PCR Purification Kit (Favorgen, Ping Pung, Taiwan) following manufacturer’s instructions. The three purified DNA fragments were then fused using In-Fusion® HD Cloning Kit following the manufacturer’s instructions (Takara, Seoul, Korea). The resulting hybridised fragment was used to transform *S. suis* using the *comS* peptide, as previously described [5-7]. Briefly, bacteria were grown overnight in THB supplemented with 5% pork serum. The culture was then diluted 1:10 in THB containing 5% pork serum and 10% glucose and incubated for 1 h until reach an OD_600_ of 0.04. The bacterial culture was then mixed with 1.2 µg of purified DNA fragment or genomic DNA, and 12.5 µM of ComS 13-21 (GNWGTWVEE) and raised up to 100 µl with bacterial culture. Mixture was incubated for 4 h at 37 ºC under 5% CO_2_. Finally, it was plated on THA plates supplemented with the appropriate antibiotics and incubated at 37ºC under 5% CO_2_ for 24-48 h.

## ***Co-incubation experiments***

Strains used as donor and recipients were independently incubated overnight in 3 ml of THB supplemented with specific antibiotics. 20 ml of THB cultures, started at OD_600_ of 0.05, were grown to obtain bacteria in exponential phase (OD_600_ ~ 0.4). Both strains were then mixed in various donor/acceptor ratios (10^3^/1, 10^2^/1, 10/1, 1/1, 1/10, 1/10^2^, and 1/10^3^) and centrifuged at 4,500 x g for 10 min. The resulting pellet was resuspended, and three drops of 2 µl each were distributed on blood agar plates and incubated at 37°C under 5% CO_2_ for 18-20 h. In some experiments, 150 mg/L, 50 mg/L or 0 mg/L of DNase I (Sigma-Aldrich, Darmstadt, Germany) was added to the culture medium by mating of donor and recipient cells, and the mixture was incubated during 30 min at 37°C. The bacteria were collected in 500 µl of 0.9% NaCl and spread on agar plates supplemented with selective antibiotics, then incubated at 37°C under 5% CO_2_. Selective antibiotics for *S. suis-S.* *suis* conjugation were tetracycline (10 mg/L), erythromycin (5 mg/L), and spectinomycin (100 mg/L). For *S. suis-S. pneumoniae* mating, the antibiotics tetracycline (10 mg/L), erythromycin (5 mg/L), and penicillin G (1 mg/L) were used for selection, while for *S. suis-S. agalactiae*, and *S. suis-S*. *pyogenes* matting, tetracycline (10 mg/L), erythromycin (5 mg/L), and rifampicin (100 mg/L) were used. The number of CFUs grown on plates were counted after 24-48 h. All trans-conjugants were confirmed by PCR. The conjugation rate was estimated by dividing the number of CFUs of the trans-conjugants by the CFUs of the donor bacteria present at the end of the conjugation experiments.

Individual growth cultures were initiated in THB, supplemented with 5% of sheep blood, when necessary, at an initial OD_600_ of 0.05 and grown to an OD_600_ of 0.4. Donor and recipient strains were co-incubated at 1:1 ratio, seeded onto blood agar, and incubated overnight at 37°C under 5% CO_2_ for 24 h. Then, bacteria were resuspended in 500 µl of THB, serially diluted, and plated in THA supplemented with 5% of sheep blood and the appropriated antibiotics for both the donor and the recipient strains. Plates were incubated at 37°C under 5% CO_2_ for 24 h. CFUs were then enumerated for each donor-recipient combination. Pure cultures of each strain were incubated in parallel as growth controls. CFU counts from co-incubation assays were compared to those of the controls to assess potential growth competition effects.

**Results**

## **Analysis of MGEs in *S. suis* isolates**

The genome of 34 *S. suis* isolates PCR-positive to *erm*(B) and *tet*(O) (Ss_02, Ss_08, Ss_20, Ss_27, Ss_31, Ss_45, Ss_46, Ss_50, Ss_52, Ss_53, Ss_61, Ss_64,Ss_69, Ss_70, Ss_72, Ss_81,Ss_84, Ss_92, Ss_93, Ss_100, Ss_105, Ss_106, Ss_109, Ss_110, Ss_115, Ss_121, Ss_124, Ss_134, Ss_146, Ss_156, Ss_160, Ss_165, Ss_166, and Ss_167) was analysed. Of note that these isolates were recovered from different geographic regions in Spain and belongs to different ST and serotypes. MGEs were detected and analysed using the software ICEScreen, which has been previously used to analyse ICEs of *S. suis* [8] and allows for exhaustive identification of ICEs and IMEs, even nested elements, as well as their classification into families. A total of 149 MGEs were detected. Among them, 62 were ICEs, which belonged to 4 families: 1) Tn*5252* (75.8%), 2) Tn*1549* (14.5%), 3) Tn*GBS2* (8.1%), and 4) ICE*St3* (1.6%). Additionally, 87 were IMEs, classified into seven families according to the characteristic domain of their relaxase: 1) PF01076 (35.6%), 2) PF02486 (32.2%), 3) PF01719 (12.6%), 4) PF01719_PF00910 (8%), 5) PHA00330 (6.9%), and 6) PF13814 (4.6%). AMR genes were found in 28 ICEs, 5 defective ICEs (dICEs), one partial ICE, 24 IMEs and 6 defective IMEs (dIMEs). Notably, all IMEs and dIMEs carrying AMR genes were located within an ICE or dICE. The ICEs or dICEs harboured up to six distinct AMR gene patterns (taking into account the order and direction of the genes [3’-5’]): 1) *tet*(O)-*erm*(B) (67.6%), 2) *erm*(B)-*tet*(O) (8.8%), 3) *tet*(O)-*aadE*-*erm*(B) (8.8%), 4) *tet*(O)-*erm*(B)-*aadE*-*sat4*-*aph*(3')-III (5.9%), 5) *erm*(B)*-*tet*(O)-*erm*(B) (2.9%) and 6) *tet*(O)-*erm*(B)* (2.9%). All ICEs carrying AMR genes belong to the Tn*5252* family (ICE_Tn*5252*) and were inserted into the *rplL* (SSU0845 29.4%), *rumA* (SSU0561 29.4%), *mutT* (SSU0877 23.5%), ADP ribose pyrophophatase (SSU1262 8.8%) or NTP pyrophosphohydrolase (SSU1797 8.8%) genes (8.8%) genes. Similarly, all IMEs carrying AMR genes belong to the PF01076 family, and they were inserted into SNF2 (encoding a putative helicase) (22/30) or PPI (peptidylprolyl isomerase) (7/30) genes, except for one IME with an undetermined insertion site. Comparative analyses of the 34 ICE_Tn*5252* carrying *tet*(O) and/or *erm*(B) AMR genes using MAUVE revealed that 22 ICEs can be grouped into 8 clusters with high sequence identity (distance > 0.85), whereas 12 ICEs displayed reduced genetic relationship (distance < 0.85). The largest cluster, corresponding to ICE_Tn*5252*_*mutT* carrying *tet*(O)-*erm*(B), was found in isolates Ss_50, Ss_84, Ss_100, Ss_110, Ss_146, and Ss_156. All these isolates belong to ST123, but the geographic origin is very diverse. Another closely related cluster, corresponding to ICE_Tn*5252*_*mutT* carrying *tet*(O)-*aadE*-*erm*(B) was detected in isolates Ss_27 and Ss_134, which belong to different STs, but are genetically related [1], and were isolated in different geographical regions. A cluster corresponding to ICE_Tn*5252*_*SSU1797* carrying *tet*(O)-*erm*(B) was detected in isolates Ss_93, Ss_20, and Ss_109. These isolates were of different and non-related STs (ST29 and ST16) and geographical origins. Two clusters of ICE_Tn*5252*_*rumA* carrying *tet*(O)-*erm*(B) were found in isolates of the same ST, but from different geographical regions: one in Ss_02, Ss_64, and Ss_81 (ST1) and the other in Ss_106 and Ss_160 (ST123). Finally, three clusters of ICE_Tn*5252*_*rplL* with different AMR gene content were detected in isolates of different STs and geographical origins. The first one was found in isolates Ss_53 and Ss_52 (ICE_Tn*5252*_*rplL* carrying *tet*(O)-*erm*(B) genes). The second cluster was found in isolates Ss_92 and Ss_167 (ICE_Tn*5252*_*rplL* carrying *tet*(O)-*erm*(B) or *tet*(O) genes, respectively. The last cluster of ICE_Tn*5252*_*rplL* carried *tet*(O)-*erm*(B)-*aadE*-*sat4*-*aph*(3’)-III and was found in isolates Ss_08 and Ss_124. A comparative analysis of representative *tet*(O)-*erm*(B) ICEs of the different clusters revealed that these ICEs share large sequences, mainly at the central region and at the 3´end, albeit in a discontinuous manner, forming a mosaic-like pattern. Notably, ICE_Tn*5252*_*rplL* from isolates Ss_167 and Ss_08 exhibited nearly identical sequences, with Ss_167 showing 100% sequence coverage and high identity (> 99%) with Ss_08. Ss_08 contained an additional insertion within the IME_PF01076_SNF2, specifically a sequence harbouring the *aadE*-*sat4*-*aph*(3’)-III.

## **Analysis of MGEs in *S. agalactiae***

The genome of the 11 *S. agalactiae* isolates carrying *tet*(O)-*erm*(B) was sequenced. These isolates belong to 5 STs: ST498 (Sa_26, Sa_37, Sa_44), ST17 (Sa_56, Sa_82, Sa_85), ST28 (Sa_48 and Sa_86), ST529 (Sa_79, and Sa_83), and ST196 (Sa_75). MGEs were identified and analysed using ICEScreen, which detected a total of 74 MGEs. Among these, 36 were ICEs from 6 families: (1) Tn*5252* (36.1%), (2) ICE*St3* (33.3%), (3) Tn*GBS2* (13.9%), (4) Tn*916* (11.1%), (5) Tn*1549* (2.8%), and (6) Tn*GBS1* (2.8%). The remaining 38 MGEs were IMEs from 6 families: (1) PF01719 (28.9%), (2) PF01076 (26.3%), (3) PF02486 (23.7%), (4) PF01076_PF02486 (7.9%), (5) PHA00330 (7.9%), and (6) PF02407 (5.3%). AMR genes were found in 18 ICEs, 6 IMEs, and 6 dIMEs. All IMEs or dIMEs with AMR genes were located within an ICE. These ICEs belonged to three families: Tn*5252* (72.2%), Tn*916* (22.2%) and ICE*St3* (5.6%), while the IMEs with AMR genes belonged to two families: PF01076 (91.7%) and PF02486 (8.3%). 6 ICEs only carried *tet*(O) and *erm*(B) (33.3%), and 3 ICEs also had *aadE*, *sat4*(partial), and *aph*(3')-III AMR genes (16.7%). Moreover, three other AMR genes patterns were found: 1) only *tet*(M) (22.2%), 2) *mrs*(D)-*mef*(A) (11.1%), and 3) *lsa*(C) (5.6%).

ICEs that contained AMR genes but lacked *tet*(O) or *erm*(B) included one ICE*St3*, found in isolate Sa_79, and an IME inserted in the *oriT* sequence which harboured the *lsa*(C) gene. Additionally, ICEs of the family Tn*916* (ICE_Tn*916*) were identified in isolates Sa_26, Sa_37, Sa_44 and Sa_86 and carried the *tet*(M) gene alone. Comparative analysis of four ICE_Tn*916* revealed that isolates Sa_26, Sa_44, Sa_37, all belonging to ST498, share an ICE of the Tn*916* family inserted into the *guaA* gene (ICE_Tn*916*_*guaA*), with *tet*(M) showing over 99% sequence identity.

The *tet*(O) and *erm*(B) genes were identified in ICEs from the Tn*5252* family and exhibited 4 distinct AMR gene patterns. These ICEs were inserted into either the *rplL* or *rumA* gene. Nearly all ICE_Tn*5252* carrying AMR genes contained *tet*(O)-*erm*(B) alone or with additional resistant genes. Exceptions included: 1) ICE_Tn*5252*_*rplL* identified in isolates Sa_56 and Sa_85 of ST17, which only carried *mef*(A)-*mrs*(D), and 2) ICE_Tn*5252*_*rplL* identified in isolates Sa_79 and Sa_83 of ST529, which showed a large variability in the AMR gene content. Notably, comparative analysis of ICE_Tn*5252* carrying *tet*(O)-*erm*(B) genes revealed regions of high identity (>95%), with conserved AMR gene arrangements. In several cases, isolates carrying identical ICEs belonged to the same ST suggesting vertical transmission of these MGEs. For example, isolates Sa_26, Sa_37, and Sa_44 of ST498, as well as isolate Sa_75 of ST196, all carried an ICE_Tn*5252*_*rplL* with *tet*(O)-*erm*(B) genes sharing >98% identity. Similarly, isolates Sa_56, Sa_82, and Sa_85, all belonging to ST17, contained an ICE_Tn*5252*_*rumA* harbouring *tet*(O)-*aadE*-*sat4*(partial)-*aph*(3')-III-*erm*(B)-*aadE,* displaying >98% sequence identity. Isolates Ss_48 and Ss_86 (ST28) carried ICE_Tn*5252*_*rumA* with *tet*(O) and *erm*(B) genes, displaying > 99% identity. Interestingly, some ICE_Tn*5252* shared considerable sequence identity and AMR gene content even when inserted at different genomic loci.

## **Analysis of transformants**

The genome of P1/7ICE115Δ*tetO* was used to transform rifampicin-resistant isolates Ss_45r and Ss_124r, which harbour ICE*Ssu_45*_Tn*5252*_*rumA* and ICE*Ssu_124*_Tn*5252*_*rplL*, respectively. This transformation yielded three transformants tetracycline-sensitive of Ss_45r (Ss_45r_Tf25, Ss_45r_Tf29, and Ss_45r_Tf33) and 3 three transformants of Ss_124r (Ss_124r_Tf5, Ss_124r_Tf8, and Ss_124r_Tf10). PCR assays were performed on these transformants to confirm recombination events. A schematic showing the location of the primers used for the analysis of ICEs in the transformants is provided in **Figure S1A,** with results for each clone detailed in **Figure S1B-C**. PCR screening revealed that clones Ss_45r_Tf25, Ss_45r_Tf33, Ss_124r_Tf5, and Ss_124r_Tf10 had both downstream- and upstream-*tet*(O) regions identical to the recipient strain (fragments *a1*/*b1* and *c*), meanwhile Ss_45r_Tf29 and Ss_124r_Tf8 had the same downstream-*tet*(O) region as the recipient, but the upstream-*tet*(O) region identical to the donor. To further test variability in the recombined ICEs, the genomes of clones Ss_45r_Tf29, Ss_45r_Tf33, and Ss_124r_Tf10 were sequenced and their ICEs analysed and compared to the original parental strains. Structural differences in the ICEs were observed in all three transformants. For Ss_45r_Tf29 (ICE*Ssu_45_Tf29*_Tn*5252*_*rumA*), the conjugation module and integrase were 99.2% homologous to the recipient strain (ICESsu_45_Tn*5252*_*rumA*), but the region surrounding the *cat* gene was 98.6% similar to donor ICE*Ssu_115*_Tn*5252*_*SSU1262*. In this clone, the IME_PF01076_SNF2 signature proteins were lost, as well as one copy of *erm*(B) gene present in the original ICE. In Ss_45r_Tf33 (ICE*Ssu_45_Tf33*_Tn*5252*_*rumA*), the conjugation module, the integrase gene, and the truncated SNF2 gene from the recipient ICE were retained, showing 99.6% of sequence identity. However, the recombination process resulted in the loss of the IME_PF01076_SNF2 relaxase, integrase, and the terminal region of the SNF2 gene. The ICE of Ss_124r_Rf10 (ICE*Ssu_124_Tf10*_Tn*5252*_*rumA*) retained 95.7% of the sequence of the original ICE with 99.2% overall identity, exhibiting only two changes: the loss of part of SNF2 and the substitution of *tet*(O) by *cat* as in ICE*Ssu_115*_Tn*5252*_*SSU1262*Δ*tetO*. The conjugative transfer of ICE*Ssu*_*124*_*Tf10*_Tn*5252_rplL* from the Ss_124r_Tf10 strain exhibited a low transconjugant rate (approximately 1.3 x10^-6^ transconjugants per donor) which was comparable to that of ICE*Ssu*_*124*_Tn*5252*_*rplL*, the original ICE of the Ss_124 strain.

**References**

1. C. Uruén, A. Fernandez, J.L. Arnal, M. Del Pozo, M.C. Amoribieta, I. de Blas, P. Jurado, J.H. Calvo, M. Gottschalk, L.D. González-Vázquez, Genomic and phenotypic analysis of invasive *Streptococcus suis* isolated in Spain reveals genetic diversification and associated virulence traits, Vet. Res. 55 (2024) 11. <https://doi.org/10.1186/s13567-024-01267-0>.

2. C. Uruén, J. Gimeno, M. Sanz, L. Fraile, C.M. Marín, J. Arenas, Invasive *Streptococcus suis* isolated in Spain contain a highly promiscuous and dynamic resistome, Front. Cell. Infect. Microbiol. 13 (2024) 1329632. <https://doi.org/10.3389/fcimb.2023.1329632>.

3. F.A. Clifton-Hadley, Studies of *Streptococcus suis* type 2 infection in pigs, Vet. Res. Commun. 8 (1984) 217-27. <https://doi.org/10.1007/bf02214715>.

4. C. García López, L. Saralegui Remón, C. Uruén García, C. Bosch Díaz, P. Jurado Romero, M. Gottschalk, J. Arenas Busto, Techniques for Genetic Manipulation, In: Arenas J, editor, *Streptococcus suis*: Methods and Protocols, New York: Springer, 2024, p. 37-49. <https://doi.org/10.1007/978-1-0716-3898-9_4>.

5. E. Zaccaria, P. van Baarlen, A. de Greeff, D.A. Morrison, H. Smith, J.M. Wells, Control of competence for DNA transformation in *Streptococcus suis* by genetically transferable pherotypes, PloS one 9 (2014) e99394. https://doi.org/10.1371/journal.pone.0099394.

6. M.L. Ferrando, A. Gussak, S. Mentink, M.F. Gutierrez, P.v. Baarlen, J.M. Wells, Active human and porcine serum induce competence for genetic transformation in the emerging zoonotic pathogen *Streptococcus suis*, Pathogens 10 (2021) 156. <https://doi.org/10.3390/pathogens10020156>.

7. Y. Zhu, W. Dong, J. Ma, Y. Zhang, Z. Pan, H. Yao, Utilization of the ComRS system for the rapid markerless deletion of chromosomal genes in *Streptococcus suis*, Future Microbiol. 14 (2019) 207-22. <https://doi.org/10.2217/fmb-2018-0279>.

8. M. Dechêne-Tempier, C. de Boisséson, P. Lucas, S. Bougeard, V. Libante, C. Marois-Créhan, S. Payot, Virulence genes, resistome and mobilome of *Streptococcus suis* strains isolated in France, Microb. Genom. 10 (2024) 001224. <https://doi.org/10.1099/mgen.0.001224>.
