# Supplementary material for: Evidence of transfer of antimicrobial resistance genes from the porcine pathogen Streptococcus suis to human clinical isolates of Streptococcus agalactiae in a major pig-producing region of Spain
Source: One Health. 2026 Mar 28;22:101396. doi: 10.1016/j.onehlt.2026.101396 (PMC13089153; doi:10.1016/j.onehlt.2026.101396)
Supplement: Supplementary Table S2 — Primers used in the study. The size of the expected PCR products and annealing temperature (TA) for each PCR reaction is also indicated. Abbreviations: bp: base pairs, TA: annealing temperature, AMR: Antimicrobial Resistance. [file mmc4.docx]

**Table S2**. Primers used in the study. The size of the expected PCR products and annealing temperature (T_A_) for each PCR reaction is also indicated.

| **Primer** | **Sequence Forward (5'-3')** | **Sequence Reverse (5'-3')** | **Reference** | **Target gene** | **PCR product (bp)** | **T_A_ (ºC)** |
| --- | --- | --- | --- | --- | --- | --- |
| **Mutant construction** | | | | | | |
| **ICE115_tetO_D** | GTTGATCGAGGGATTCCAAA | AGTCAGTCGATCACAACTATGTGATTTTCCTCCTATCAAC | This study | Downstream flank *tet*(O) | 999 | 55 |
| **ICE115_tetO_U** | AGTCTACGTACATAGTCAGTGTTCCACAAGTTAGCTTAAC | TTGTAAACGTGGATCATTTT | This study | Upstream flank *tet*(O) | 1110 | 55 |
| **CAM** | ATAGTTGTGATCGACTGACTAAGGGATCCGGCACCTATCT | ACTGACTATGTACGTAGACTTGCCCGGGGATCCTCCGATA | This study | *cat* | 1156 | 55 |
| **AMR gene detection and co-localization** | | | | | | |
| **tetO** | AACTTAGGCATTCTGGCTCAC | TCCCACTGTTCCATATCGTCA | [1] | *tet*(O) | 515 | 61 |
| **ermB** | GCAGTGACTAATCTTATGACTTTT | CTGTGGTATGGCGGGTAAGT | [1] | *erm*(B) | 621 | 63 |
| **Identification of *Streptococcus* species** | | | | | | |
| **JP4-JP5** | GCAGCGTATTCTGTCAAACG | CCATGGACAGATAAAGATGG | [2] | *gdh* | 688 | 55 |
| **Spnm** | CAACCGTACAGAATGAAGCGG | TTATTCGTGCAATACTCGTGCG | [3] | *lytA* | 319 | 53 |
| **Sag** | CTGAAAATTACAATTAGTATGGAAGA | CCACATTAGCATGTTCAAGC | This study | *mecA* | 634 | 55 |
| **Spyg** | AAAGACCGCCTTAACCACCT | TGGCAAGGTAAACTTCTAAAGCA | [4] | *spy* | 407 | 55 |
| **SPC** | GCAGGTCGATTTTCGTTCGT | ATGCAAGGGTTTATTGTTTTCTAA | [5] | Spectinomycin resistance cassette | 1159 | 55 |
| **Assessing recombination** | | | | | | |
| **ICE45_rec_D** | GGACAGGACTTTGAGGTCGT | CTATCACTGGGTAAATAACT | This study and [6] | Downstream flank of *tet*(O)/*cat* | * | 52 |
| **ICE124_rec_D** | TTCAGGGAGTATTGGACACT | ICE45_tetO_D-Rev | This study | Downstream flank of *tet*(O)/*cat* | * | 55 |
| **ICE_rec_U** | ATGAAGAAACAGGATTTTAAGGTGT | ermB-Rev | This study | Upstream flank of *tet*(O)/*cat* | * | 52 |

*Molecular size of the expected fragment varies due to the template.

Abbreviations: bp: base pairs, T_A_: annealing temperature, AMR: Antimicrobial Resistance.

**References**

1. Malhotra-Kumar, S., et al., *Multiplex PCR for simultaneous detection of macrolide and tetracycline resistance determinants in streptococci.* Antimicrobial agents and chemotherapy, 2005. **49**(11): p. 4798-4800.

2. Okwumabua, O., M. O'Connor, and E. Shull, *A polymerase chain reaction (PCR) assay specific for Streptococcus suis based on the gene encoding the glutamate dehydrogenase.* FEMS microbiology letters, 2003. **218**(1): p. 79-84.

3. Nagai, K., et al., *Evaluation of PCR primers to screen for Streptococcus pneumoniae isolates and β-lactam resistance, and to detect common macrolide resistance determinants.* Journal of Antimicrobial Chemotherapy, 2001. **48**(6): p. 915-918.

4. Liu, D., et al., *Rapid identification of Streptococcus pyogenes with PCR primers from a putative transcriptional regulator gene.* Research in microbiology, 2005. **156**(4): p. 564-567.

5. Arenas, J., et al., *In vivo transcriptomes of Streptococcus suis reveal genes required for niche-specific adaptation and pathogenesis.* Virulence, 2019. **10**(1): p. 334-351.

6. Libante, V., et al., *Chromosomal conjugative and mobilizable elements in Streptococcus suis: major actors in the spreading of antimicrobial resistance and bacteriocin synthesis genes.* Pathogens, 2019. **9**(1): p. 22.
