# Supplementary material for: Evidence of transfer of antimicrobial resistance genes from the porcine pathogen Streptococcus suis to human clinical isolates of Streptococcus agalactiae in a major pig-producing region of Spain
Source: One Health. 2026 Mar 28;22:101396. doi: 10.1016/j.onehlt.2026.101396 (PMC13089153; doi:10.1016/j.onehlt.2026.101396)
Supplement: Supplementary Table S3 — Molecular sizes (kb) obtained by PCR for the co-localization of tet(O) and erm(B) genes in 91 S. suis isolates and 11 S. agalactiae isolates. [file mmc5.docx]

**Table S3**. Molecular sizes (kb) obtained by PCR for the co-localization of *tet*(O) and *erm*(B) genes in 91 *S. suis* isolates and 11 *S. agalactiae isolates*.

| **Distance (kb)** | ***S. suis*** | | ***S. agalactiae*** | |
| --- | --- | --- | --- | --- |
|  | **Percentage (%)** | **Isolates** | **Percentage (%)** | **Isolates** |
| **2** | 2.2 | Ss_03, Ss_99 | 0 |  |
| **3** | 1.1 | Ss_104 | 0 |  |
| **3.5** | 8.8 | Ss_20, Ss_93, Ss_109, Ss_166, Ss_35, Ss_97, Ss_125, Ss_171 | 0 |  |
| **5** | 2.2 | Ss_11, Ss_62 | 0 |  |
| **6** | 1.1 | Ss_115 | 0 |  |
| **8** | 1.1 | Ss_84 | 0 |  |
| **9** | 2.2 | Ss_105, Ss_160 | 0 |  |
| **10** | 40.7 | Ss_04, Ss_08, Ss_13, Ss_15, Ss_16, Ss_17, Ss_18, Ss_26, Ss_34, Ss_37, Ss_44, Ss_45, Ss_46, Ss_49, Ss_50, Ss_54, Ss_66, Ss_72, Ss_84, Ss_86, Ss_88, Ss_100, Ss_101, Ss_106, Ss_110, Ss_117, Ss_124, Ss_127, Ss_132, Ss_133, Ss_138, Ss_146, Ss_154, Ss_156, Ss_159, Ss_163, Ss_170 | 45.5 | Sa_26, Sa_37, Sa_44, Sa_75, Sa_86 |
| **12** | 13.2 | Ss_09, Ss_70, Ss_73, Ss_77, Ss_79, Ss_82, Ss_85, Ss_92, Ss_102, Ss_155, Ss_157, Ss_167 | 9.1 | Sa_48 |
| **13** | 1.1 | Ss_134 | 27.3 | Sa_56, Sa_82, Sa_85 |
| **14** | 1.1 | Ss_69 | 0 |  |
| **15** | 4.4 | Ss_31, Ss_52, Ss_53, Ss_61 | 0 |  |
| **16** | 4.4 | Ss_02, Ss_27, Ss_64, Ss_81 | 0 |  |
| **17** | 2.2 | Ss_38, Ss_60 | 0 |  |
| **20** | 1.1 | Ss_121 | 0 |  |
| **Negative*** | 13.2 | Ss_10, Ss_25, Ss_28, Ss_114, Ss_123, Ss_126, Ss_128, Ss_129, Ss_131, Ss_140, Ss_141, Ss_165 | 18.2 | Sa_79, Sa_83 |

* Three replicates were performed with an elongation time of 20 minutes in each cycle.
