# Supplementary material for: Evidence of transfer of antimicrobial resistance genes from the porcine pathogen Streptococcus suis to human clinical isolates of Streptococcus agalactiae in a major pig-producing region of Spain
Source: One Health. 2026 Mar 28;22:101396. doi: 10.1016/j.onehlt.2026.101396 (PMC13089153; doi:10.1016/j.onehlt.2026.101396)
Supplement: Supplementary Table S5 — Prevalence of antimicrobial resistance to different antibiotics in isolates of S. pneumoniae, S. agalactiae, and S. pyogenes from human patients at Miguel Servet University (Zaragoza, Spain) between 2019 and 2021. [file mmc7.docx]

**Table S5**. Prevalence of antimicrobial resistance to different antibiotics in isolates of *S. pneumoniae, S. agalactiae,* and *S. pyogenes* from human patients at Miguel Servet University (Zaragoza, Spain) between 2019 to 2021.

| **Specie** | **Isolates** | **Resistant isolates (%)** | | | |
| --- | --- | --- | --- | --- | --- |
|  |  | **Erythromycin** | **Clindamycin** | **Tetracycline** | **Penicillin** |
| ***S. pneumoniae*** | 529 | 27.5 | 22.1 | 21.6 | 16.3 |
| ***S. agalactiae*** | 1,497 | 27.5 | 24 | 59.1 | 0 |
| ***S. pyogenes*** | 362 | 4.5 | 3.9 | 11.5 | 0 |
